# Compound Danshen Dripping Pills Prevented Leptin Deficiency-Induced Hepatic ER Stress, Stimulated Autophagy, and Improved Insulin Resistance of ob/ob Mice

**DOI:** 10.1155/2020/5368657

**Published:** 2020-07-03

**Authors:** Yanan Shi, Dan Liu, Jihong Yuan, Lihui Yan, Zhenfeng Zhan, Da Pan, Laixiang Lin, Biao Mu

**Affiliations:** ^1^NHC Key Laboratory of Hormones and Development (Tianjin Medical University), Tianjin Key Laboratory of Metabolic Diseases, Tianjin Medical University Chu Hsien-I Memorial Hospital & Tianjin Institute of Endocrinology, Tianjin 300134, China; ^2^Cargill Feed (Tianjin) Co., Ltd., No. 6 Huifeng Road, Wuqing Developing Zone, Tianjin 301700, China

## Abstract

Compound Danshen dripping pills (CDDP) is widely used for the treatment of coronary arteriosclerosis and ischemic heart diseases for decades of years. In our study, we interestingly discovered the effects and mechanism of CDDP on insulin resistance that increase the risk factor of cardiovascular diseases. Effects of CDDP on fasting blood glucose, the insulin tolerance test (ITT), the oral glucose tolerance test (OGTT), hepatic function, and underlying mechanism were analyzed in ob/ob mice. CDDP was found improving the impaired insulin signal sensitivity of ob/ob mice by ameliorating insulin and glucose tolerance, improving hepatic phosphorylation of the insulin receptor substrate-1 on Ser 307 (pIRS1) of ob/ob mice, and restoring hepatic function by decreasing serum ALT and AST, which increased in ob/ob mice serum. Decreasing hepatic phosphorylation of pancreatic ER kinase (PERK) and inositol-requiring enzyme-1 (IRE1) regulating hepatic ER stress in the liver of ob/ob mice were increased by CDDP. Furthermore, CDDP was also found stimulating ob/ob mice hepatic autophagy by increasing the expression of Beclin1 and LC3B, while decreasing P62 expression. Our study discovered an important role of CDDP on improving ob/ob mice insulin resistance and liver function probably through relieving hepatic ER stress and stimulating hepatic autophagy, which would broaden the application value and provide more benefits for treating cardiovascular patients. This trial is registered with NCT01659580.

## 1. Introduction

Compound Danshen dripping pills (CDDP, also known as Dantonic®) launched in 1995 by the China Food and Drug Administration (CFDA) for the treatment of angina pectoris (AP) has a rich clinical history for the past 24 years with an estimated more than 450 million patients in China [1], and it has been approved to enter the phase III (NCT01659580) clinical trial by the US Food and Drug Administration in 2016 [2]. Nowadays, CDDP is widely applied for the treatment of cardiovascular events such as AP, ischemic heart diseases, and coronary arteriosclerosis in China [3, 4], and CDDP is also marketed as a prescription or nonprescription drug for the prevention and treatment of ischemic heart diseases in Singapore, Canada, South Korea, Mongolia, and Vietnam [5]. Modern pharmacological studies have reported that CDDP has also antioxidative, anti-inflammatory, and endothelium-protective effects, and inhibition of atherosclerotic plaque formation and neointimal hyperplasia. CDDP is composed of the mixed extracts of *S. miltiorrhiza*, notoginseng, and borneol. Among them, *S. miltiorrhiza* extracts have positive effects on antiperoxidative damage, myocardial protection, myocardial energy metabolism improvement, and antiplatelet aggregation activities [3, 6]. In brief, CDDP was used and researched mainly based on cardiovascular protection effects. No more information was developed based on its antioxidative effects; also, no more research studies on CDDP were performed on insulin resistance, which is closely related with cardiovascular injury, and on important pathogenic factors of cardiovascular diseases.

Cardiovascular disease is the leading cause of mortality among individuals with diabetes mellitus, and >50% of patients will die not only from a cardiovascular event, especially coronary artery disease, but also from stroke and peripheral vascular disease. Patients with T1DM develop insulin resistance in the months after diabetes mellitus diagnosis, and patients with T2DM typically develop insulin resistance before hyperglycaemia occurs. Insulin resistance and hyperglycaemia, in turn, further increase the risk of adverse cardiovascular events [7].

Resistance of target tissues mainly the liver, adipose, and skeletal muscle to insulin is the major pathophysiological event contributing to the development of type 2 diabetes mellitus. Resistance of target tissues to insulin was regulated by extremely complex mechanisms; endoplasmic reticulum (ER) stress is thought to play a crucial role in the development of insulin resistance and the pathogenesis of diabetes, and it is evident in obese animals and humans [8–12]. As an intracellular organelle, ER coordinates the synthesis, folding, and trafficking of proteins. During stress, unfolded and misfolded proteins accumulate in the ER and initiate an adaptive response known as the unfolded protein response (UPR) via three ER transmembrane proteins, such as the PKR-like ER kinase (PERK), inositol requiring enzyme 1 (IRE1), and activating transcription factor (ATF) 6 [13]. Timely clearance of unfolded and misfolded proteins means relief of the pathological state of organism; autophagy plays an essential role in the clearance of aggregated toxic proteins and degradation of the damaged organelles, and it is an intracellular catabolic process with an essential function in the maintenance of cellular and energy homeostasis [14] and has been reported to be associated with many metabolic disorders, impairment of autophagy which showed an accumulation of altered and deformed mitochondria and of lipid droplets, and an increased number of ubiquitinated protein aggregates in the liver, adipose tissue, as well as impaired glucose tolerance in the muscle [15, 16]. During the autophagy process, an autophagosome is formed within a double-membrane-bound vesicle surrounding isolate-targeted or nonspecific materials and subsequently fused with endolysosomal vesicles forming autolysosome, which further leads to enzymatic degradation and recycling of the sequestered substrates [17] and the process of autophagy depending on series proteins binding and functioning at different stages, such as Beclin1 for nucleation of the phagophore, microtubule-associated protein 1 light chain 3 (LC3) for characteristic signature of autophagic membranes, and P62 for clearance of protein aggregates [18].

Based on the current usage and situation, we interestingly discovered the effects and mechanism of CDDP as a cardiovascular protection drug on insulin resistance of ob/ob mice, which would broaden the application value and provide more benefits for treating cardiovascular patients.

## 2. Materials and Methods

### 2.1. Drugs

CDDP (27 mg/pill, lot 100605) was supplied by Tianjin Tasly Pharmaceutical Co., Ltd., and it was dissolved into sterile deionized water (ddH_2_O).

### 2.2. Experimental Animals

The animal experimental protocol was conducted according to the guidelines for the care and use of laboratory animals approved by the Tianjin Medical University's Institutional Animal Care and Use Committee. Male C57BL/6 and leptin deficient ob/ob mice (Model Animal Research Center of Nanjing University, China) were maintained in a temperature-controlled room (25°C) on a 12-hour light-dark cycle; 6 mice in each group were used. Groups for experiment 1 to assess the metabolic characters of ob/ob mice are as follows: (1) C57BL/6 mice of 8 weeks were used as the control group and (2) ob/ob mice of 8 weeks were used as the experimental group. The mice were sacrificed 2 day after the OGTT test. Groups for experiment 2 to assess the effect of CDDP on ob/ob mice are as follows: (1) 6-weeks ob/ob mice which received 200 *μ*l CDDP by daily gavage (42 mg/Kg) for 30 days were used as the treated group. (2) 6-weeks ob/ob mice which received 200 *μ*l ddH_2_O by daily gavage for 30 days were used as the experimental group. OGTT of mice fasting for 16 h was measured on day 20 of gavage, and ITT of mice fasting for 4 h was measured on day 28 of gavage. The fasting blood glucose of mice for both the experiments after fasting for 16 h was measured before sacrifice; the livers were dissected and snap frozen in liquid nitrogen. It is then stored at −80°C.

### 2.3. Measurement of Fasting Blood Glucose

Mice were fasted for 16 hours; then, blood glucose was recorded from the tail vein using a blood glucose reader (ONETOUCH, UltraVue, Johnson).

### 2.4. Measurement of ITT

After 4 hours fasting, insulin (2 IU/kg, Humulin R, Eli Lilly Japan K. K.) was injected into mice intraperitoneally, and blood samples were collected from the tail vein at the time point indicated and analyzed.

### 2.5. Measurement of OGTT

Mice were fasted overnight (16 h), and glucose challenge was initiated with a gavage of glucose (2 g/kg). Glucose levels were determined in blood drops obtained by clipping the tail of the mice immediately before the glucose challenge, as well as at 30, 60, and 120 min intervals.

### 2.6. Detection of the Liver Function

Mice blood was collected and placed in room temperature for 2 hours. It was then centrifuged (3000 rpm) for 20 minutes to obtain serum. Serum aspartate aminotransferase (AST), alanine aminotransferase (ALT), and alkaline phosphatase (ALP) were analyzed by an automatic biochemical analyzer (Roche, Modular P800).

### 2.7. Western Blotting Analysis

Western blotting was performed as previously described. Protein lysates were subjected to SDS-PAGE, transferred to hybond-PVDF membranes, and then, they were incubated with specific primary antibodies against phospho-PERK (T980), phospho-IRS1 (S307), IRS1 (cell signal technology, USA), PERK, IRE1, phospho-IRE1 (S724), P62 (Abcam, USA), Beclin1 (Immuno Way Biotechnology Company, USA), LC3B (sigma, USA), and GAPDH (Immuno Way Biotechnology Company, USA) overnight at 4°C. After washing, the membrane was incubated with anti-mouse or anti-rabbit secondary antibodies (ZSGB, China) for 2 h at room temperature. ECL was purchased from Millipore.

### 2.8. Statistical Analysis

The data were analyzed as mean ± SD. Statistical analyses were performed by ANOVA, and the means were compared by Fisher's protected least-significant difference using StatView software from SAS Institute Inc. (Cary, NC). A *p* value <0.05 was considered statistically significant.

## 3. Results

### 3.1. CDDP Improved Glucose Tolerance and Insulin Resistance in ob/ob Mice

**Figure 1:**
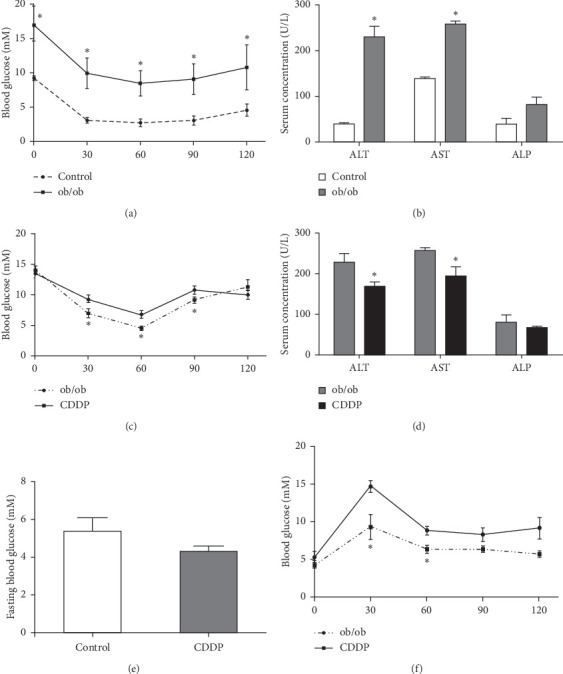
The effect of CDDP on insulin resistance and the liver function. Insulin tolerance (a) and serum AST, ALT, and ALP (b) were measured between the control mice and ob/ob mice. The insulin tolerance test (c), serum AST, ALT, and ALP (d), fasting blood glucose (e), and glucose tolerance test (f) were performed between two groups. The blood glucose was measured and compared at the indicated time point (a), (c), and (f). ^*∗*^*p* < 0.05, compared to the control mice (a) and (b) and compared to the ob/ob mice (c)–(f)).

In our study, male ob/ob mice of 6 weeks maintained a higher level of blood glucose within 2 hours after intraperitoneal injection of insulin (Figure 1(a)) and a significant increase of the serum ALT and AST level (Figure 1(b)). The result of the insulin tolerance test showed a significantly lower blood glucose level at 30, 60, and 90 minute time points after an intraperitoneal injection of insulin in mice which received CDDP compared to the control group (Figure 1(c)). Meanwhile, CDDP treatment ameliorated the level of serum ALT and AST significantly (Figure1(d)); although CDDP showed little effect on reducing fasting blood glucose (Figure 1(e)), it significantly decreased the blood glucose level at 30 and 60 minute time point after the oral glucose challenge (Figure 1(f)). Our results indicated that CDDP contributed to improving insulin sensitivity and glucose tolerance, and it showed benefit on ameliorating insulin resistance and improving hepatic dysfunction in ob/ob mice.

### 3.2. CDDP Reduced Hepatic Phosphorylation of IRS1 at Ser 307 in the Liver of ob/ob Mice

**Figure 2:**
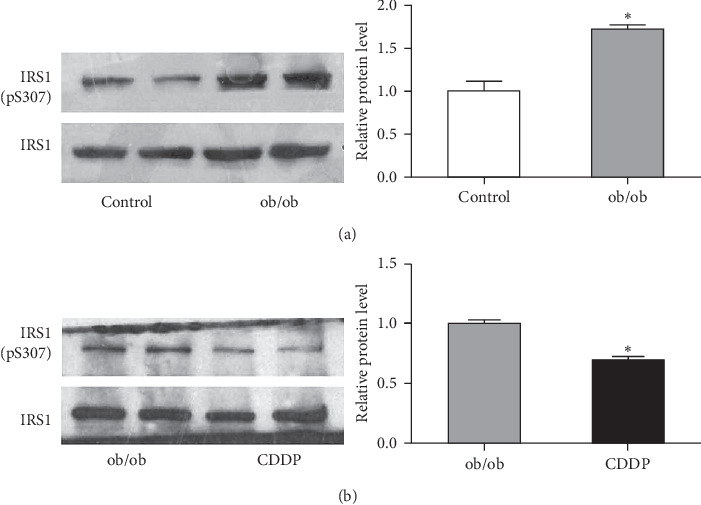
CDDP improved hepatic insulin signal sensitivity. (a) The insulin signaling marker IRS1 Ser307 phosphorylation in the liver was compared between the control mice and ob/ob mice. (b) CDDP-regulated hepatic phosphorylation of IRS1 serine 307 was of ob/ob mice. ^*∗*^*p* < 0.05, compared to the control mice for (a) and compared to ob/ob mice for (b).

We further investigated the hepatic insulin receptor signal and found an elevated phosphorylation of hepatic IRS1. Serine 307 in the liver of ob/ob mice was found (Figure 2(a)); meanwhile, CDDP represented a significant inhibition of phosphorylated IRS1 serine 307 in the liver compared with the control ob/ob mice (Figure 2(b)). It suggested that CDDP stimulates the hepatic insulin signal sensitivity, and therefore, it improves the insulin resistance.

### 3.3. CDDP Addition Improved Hepatic ER Stress of ob/ob Mice

**Figure 3:**
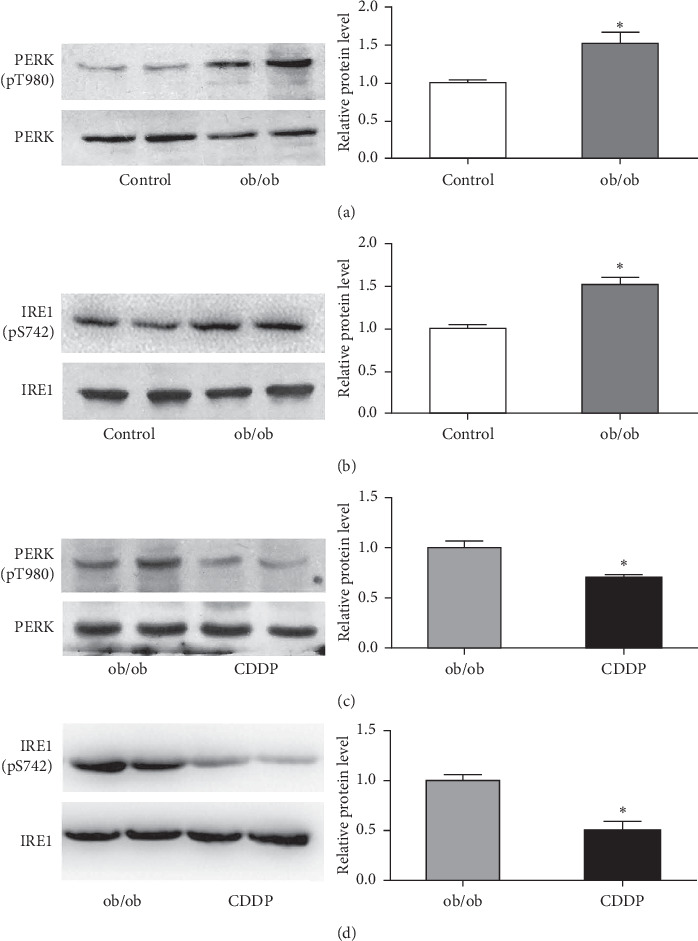
CDDP reduced hepatic ER stress induced in the liver of ob/ob mice. The expressions of ER stress markers including phosphorylated PERK Tyr980 and IRE1 Ser742 were measured and compared between the control and ob/ob mice (a) and (b) and between ob/ob and CDDP-treated mice (c) and (d). ^*∗*^*p* < 0.05, compared to the control mice for (a) and (b) and compared to ob/ob mice for (c) and (d).

ob/ob mice showed an increasing hepatic ER stress with the higher level of phosphorylation of PERK on T980 and IRE1 on S742 compared to the lean control mice (Figures 3(a) and 3(b)). CDDP-treated ob/ob mice showed a significant decrease of hepatic phosphorylation of PERK and IRE1 (Figures 3(c) and 3(d)), which indicated that CDDP attenuate hepatic ER stress in ob/ob mice.

### 3.4. CDDP Improved Impaired Hepatic Autophagy of ob/ob Mice

**Figure 4:**
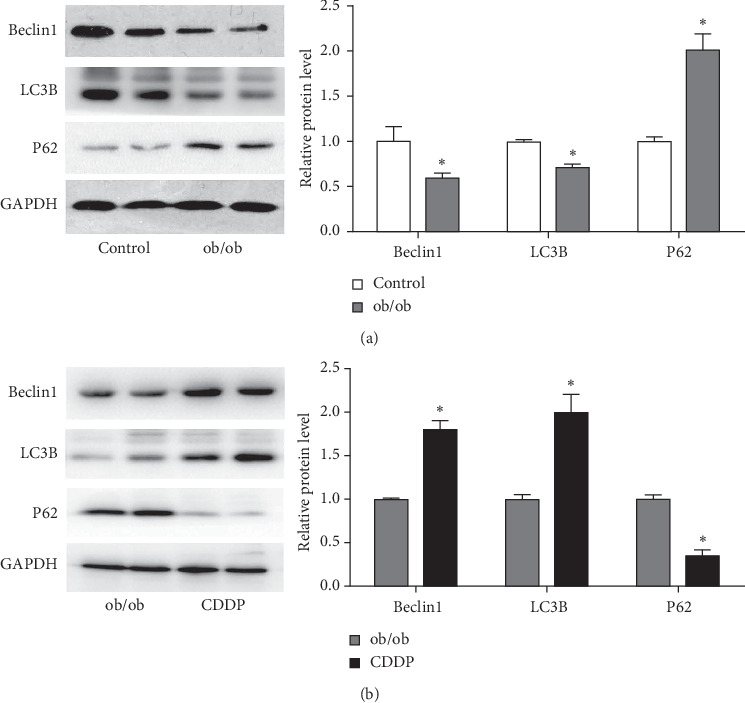
CDDP increased hepatic autophagy inhibited in the liver of ob/ob mice. Hepatic expressions of Beclin 1, LC3B, and P62 were measured and compared between the control and ob/ob mice (a) and between ob/ob and CDDP-treated mice (b). ^*∗*^*p* < 0.05, compared to the control mice for (a) and (b) and compared to ob/ob mice for (c) and (d).

A reduction of autophagy by decreasing Beclin1 and LC3B expression but increasing P62 expression in the liver of ob/ob mice was discovered (Figure 4(a)). We further investigated the effect of CDDP on hepatic autophagy of ob/ob mice and found that CDDP dramatically stimulated the hepatic expression of Beclin1 and LC3B, whereas it inhibited P62 expression (Figure 4(b)), which demonstrated that CDDP has a strong ability to stimulate hepatic autophagy flux to clean up the damage protein and protect the liver from oxidative stress damage and improve insulin resistance.

## 4. Discussion

In our study, we found significant effects of CDDP on improving glucose tolerance, insulin resistance, serum ALT, and AST in the leptin deficient ob/ob mice, but the body weight of mice was not impacted by CDDP (data were not published), which indicated a potential therapeutic role of CDDP on ameliorating insulin resistance and improving the hepatic function of ob/ob mice. Among lots of compound herb drugs, Erchen decoction and Linguizhugan decoction were also found improving insulin resistance by inhibiting IRS-1Ser307 phosphorylation in vivo and in vitro [19]; like CDDP, they also contain thousands of active ingredients from natural plants whose special therapeutic or protection effects have not been analyzed completely, but they might provide more clinical references and clinical application based on the gradual invitation of their underlying mechanism.

Furthermore, we discovered that CDDP showed a strong effect on improving hepatic insulin sensitivity by reducing serine residue 307 phosphorylation of IRS1; meanwhile, CDDP also improved ob/ob mice hepatic ER stress by decreasing p-PERK and p-IRE; reduction of ER stress has been shown to protect cells from ER stress, significantly improved insulin resistance, and markedly ameliorated glucose tolerance in the liver of obese diabetic mice [20]. ER stress is found to suppress insulin receptor signaling through the hyperactivation of c-Jun N-terminal kinase (JNK) [9]. JNK negatively regulated insulin signaling through serine phosphorylation of residue 307 within IRS1 in the liver and adipose tissues, and phosphorylation of Ser307 of IRS-1 by JNK has been found to be associated with decreased insulin-stimulated IRS-1 Tyr phosphorylation and insulin resistance [21]. Therefore, CDDP-attenuated ER stress and Ser307 phosphorylation of IRS-1 might contribute to improve insulin sensitivity. Based on these, a small molecule compound targeting ER stress to relieve T2MD also reported that chemical chaperones 4-phenyl butyric acid (PBA) and taurine-conjugated ursodeoxycholic acid (TUDCA) enhanced the adaptive capacity of the ER and acts as a potent antidiabetic modality with potential application in the treatment of T2DM [22]. ER stress was also reported mediating the liver injury by increasing the serum activity of ALT, AST, LDH, and damaged hepatocytes exhibiting ballooning, degeneration, and necrosis, and FXR ameliorates the liver injury induced by ER stress [23], that means improvement of ER stress could ameliorate the liver injury. These revealed the clinical value of CDDP on alleviating insulin resistance and the liver injury based on its mechanism of reducing ER stress and phosphorylation of IRS1.

We also found that CDDP showed a recovery of autophagy by increasing LC3 and Beclin1, while decreasing P62 expression in the liver of ob/ob mice. As important proteins for autophagy, downregulation of LC3, Beclin 1, Atg5, and Atg7 protein levels and elevation of P62 expression in the liver were also reported associated with insulin resistance, and the hepatic lipid metabolism of mice received a high fat diet [24]. Actually, dysregulation of autophagy also contribute to the development of metabolic disorders, including insulin resistance, diabetes mellitus, obesity, atherosclerosis, and osteoporosis [25, 26]; autophagy also protects the insulin target organs (liver, adipose tissue, skeletal muscle, and kidneys) from oxidative stress damage derived by hyperglycaemia [27]. Based on these, our results further recovered CDDP-ameliorated liver autophagy, which also was benefited to the improvement of insulin resistance and recovery of hepatic function.

## 5. Conclusions

In conclusion, as a Chinese traditional herbal medicine, CDDP has been widely used in China for patients with cardiovascular diseases. Our study discovered its important role on improving ob/ob mice insulin resistance and the liver function probably through both relieving hepatic ER stress and stimulating hepatic autophagy, which would broaden the application value and confer CDDP more superiorly for treating cardiovascular patients with T2MD or other damage correlated with ER stress or autophagy disruption.

## Data Availability

The data used to support the findings of this study are available from the corresponding author upon request.

## References

[1] Li X. Y. (2016). The Chinese experts suggestions on the clinical practice of T89. *Chinese Journal of Geriatrics Research*.

[2] Tasly Pharmaceuticals (2016). Phase III trial of Dantonic® (T89) capsule to prevent and treat stable angina. https://www.clinicaltrials.gov/ct2/show/NCT01659580.

[3] Li X.-Y. (2017). Recommendations on the clinical use of compound Danshen dripping pills. *Chinese Medical Journal*.

[4] Liao W., Ma X., Li J. (2019). A review of the mechanism of action of Dantonic for the treatment of chronic stable angina. *Biomedicine & Pharmacotherapy*.

[5] Sun H., Guo Z. X., Li L. Y. (2017). Case study of compound traditional Chinese medicine globalization. *Modernization of Traditional Chinese Medicine Matera Medica-World Science and Technology*.

[6] Zhang B. L., Gao X. M., Shang H. C. (2003). Study on pharmaceutical matters and functional mechanisms of complex prescriptions of radix *Salvia miltiorrhiza*. *World Science Technology-Modernization of Traditional Chinese Medicine*.

[7] Markku L., Kuusisto J. (2014). Insulin resistance and hyperglycaemia in cardiovascular disease development. *Nature Reviews Endocrinology*.

[8] Ozcan U., Cao Q., Yilmaz E. (2004). Endoplasmic reticulum stress links obesity, insulin action, and type 2 diabetes. *Science*.

[9] Ozcan U., Yilmaz E., Ozcan L. (2006). Chemical chaperones reduce ER stress and restore glucose homeostasis in a mouse model of type 2 diabetes. *Science*.

[10] Sharma N. K., Das S. K., Mondal A. K. (2008). Endoplasmic reticulum stress markers are associated with obesity in nondiabetic subjects. *The Journal of Clinical Endocrinology & Metabolism*.

[11] Gregor M. F., Yang L., Fabbrini E. (2009). Endoplasmic reticulum stress is reduced in tissues of obese subjects after weight loss. *Diabetes*.

[12] Hotamisligil G. S. (2010). Endoplasmic reticulum stress and the inflammatory basis of metabolic disease. *Cell*.

[13] Ron D., Walter P. (2007). Signal integration in the endoplasmic reticulum unfolded protein response. *Nature Reviews Molecular Cell Biology*.

[14] Parzych K. R., Klionsky D. J. (2014). An overview of autophagy: morphology, mechanism, and regulation. *Antioxidants & Redox Signaling*.

[15] Zhang Y., Goldman S., Baerga R., Zhao Y., Komatsu M., Jin S. (2009). Adipose-specific deletion of autophagy-related gene 7 (atg7) in mice reveals a role in adipogenesis. *Proceedings of the National Academy of Sciences*.

[16] Kim K. H., Jeong Y. T., Oh H. (2013). Autophagy deficiency leads to protection from obesity and insulin resistance by inducing fgf21 as a mitokine. *Nature Medicine*.

[17] He C., Klionsky D. J. (2009). Regulation mechanisms and signaling pathways of autophagy. *Annual Review of Genetics*.

[18] Kroemer G., Mariño G., Levine B. (2010). Autophagy and the integrated stress response. *Molecular Cell*.

[19] Ivan D., Zvulun E. (2018). Mechanism and medical implications of mammalian autophagy. *Nature Reviews Molecular Cell Biology*.

[20] Zhang H. C., Ta N., Chen P. M. (2017). Erchen decoction and linguizhugan decoction ameliorate hepatic insulin resistance by inhibiting IRS-1ser307 phosphorylation in vivo and in vitro. *Evidence-Based Complementary and Alternative Medicine*.

[21] Urano F., Wang X., Bertolotti A. (2000). Coupling of stress in the ER to activation of JNK protein kinases by transmembrane protein kinase IRE1. *Science*.

[22] Aguirre V., Uchida T., Yenush L., Davis R., White M. F. (2000). The c-jun NH2-terminal kinase promotes insulin resistance during association with insulin receptor substrate-1 and phosphorylation of ser307. *Journal of Biological Chemistry*.

[23] Umut Ö., Erkan Y., Lale Ö. (2006). Hotamisligil chemical chaperones reduce ER stress and restore glucose homeostasis in a mouse model of type 2 diabetes. *Science*.

[24] Han C. Y., Rho H. S., Kim A. (2018). FXR inhibits endoplasmic reticulum stress-induced NLRP3 inflammasome in hepatocytes and ameliorates liver injury. *Cell Reports*.

[25] Liu H.-Y., Han J., Cao S. Y. (2009). Hepatic autophagy is suppressed in the presence of insulin resistance and hyperinsulinemia. *Journal of Biological Chemistry*.

[26] Rocchi A., He C. (2015). Emerging roles of autophagy in metabolism and metabolic disorders. *Frontiers in Biology*.

[27] Kim K. H., Lee M.-S. (2014). Autophagy: a key player in cellular and body metabolism. *Nature Reviews Endocrinology*.

